# Nanosized Anisotropic Sm–Fe–N Particles with Metastable TbCu7-Type Structures Prepared by an Induction Thermal Plasma Process

**DOI:** 10.3390/nano15131045

**Published:** 2025-07-05

**Authors:** Yusuke Hirayama, Jian Wang, Masaya Shigeta, Shunsuke Tsurumi, Makoto Sugimoto, Zheng Liu, Kenta Takagi, Kimihiro Ozaki

**Affiliations:** 1 National Institute of Advanced Industrial Science and Technology, 4-205, Sakurazaka, Moriyama, Nagoya 463-8560, Japan; wang.jian86@aist.go.jp (J.W.); liu-z@aist.go.jp (Z.L.); k-takagi@aist.go.jp (K.T.); k-ozaki@aist.go.jp (K.O.); 2 Department of Mechanical Systems Engineering, Graduate School of Engineering, Tohoku University, 6-6-01, Aramaki Aza Aoba, Aoba, Sendai 980-8579, Japan; shigeta@tohoku.ac.jp (M.S.); shunsuke.tsurumi.s4@dc.tohoku.ac.jp (S.T.); msugimoto@tohoku.ac.jp (M.S.)

**Keywords:** Sm–Fe–N, TbCu_7_, permanent magnet, thermal plasma, anisotropic, co-condensation

## Abstract

TbCu_7_-type Sm-based compounds can be produced in bulk and potentially surpass Nd_2_Fe_14_B as permanent magnets. However, as the processes to prepare anisotropic magnetic particles are limited, the full potential of TbCu_7_-type Sm-based compounds cannot be exploited. In this study, metastable TbCu_7_-type phases of anisotropic Sm–Fe–N ultrafine particles were prepared using the low-oxygen induction thermal plasma (LO-ITP) process. X-ray diffraction analysis revealed that the obtained TbCu_7_-type Sm–Fe alloy nanoparticles exhibited a c/a value of 0.8419, with an Fe/Sm atomic ratio of ~8.5. After nitrogenation, the obtained Sm–Fe–N nanoparticles were aligned under an external magnetic field, indicating that each alloy particle exhibited anisotropic magnetic properties. A substantially high degree of alignment of 91 ± 2% was achieved, quantitatively estimated via pole figure measurements. Numerical analysis following Sm–Fe nanoparticle formation showed that, compared with Fe condensation, Sm condensation persisted even at low temperatures, because of a significant difference in vapor pressure between Sm and Fe. Though this led to a relatively large compositional distribution of Sm within particles with a Sm concentration of 9–12 at%, the preparation of single-phase TbCu_7_-type Sm–Fe–N particles could be facilitated by optimizing several parameters during the LO-ITP process.

## 1. Introduction

For the purpose of achieving carbon neutrality, the development of electric vehicles and aircraft is attracting considerable attention. High-output, high-efficiency motors are essential for realizing such types of electrification, necessitating the development of permanent magnets that can withstand high temperatures. Currently, high-efficiency interior permanent magnet motors use neodymium magnets composed mainly of Nd_2_Fe_14_B [1,2]. However, neodymium magnets are limited by their relatively low heat resistance. For instance, at approximately 150 °C, which is the operating temperature of electric vehicle motors, the performance of a permanent magnet deteriorates significantly. Therefore, by introducing numerous heavy rare-earth elements such as Dy and Tb to improve magnetic anisotropy [3,4], the required coercivity of 0.8 MA/m at approximately 150 °C can be achieved [5]. However, the use of these heavy rare-earth elements must be reduced or eliminated owing to resource concerns and decreased magnetization. Recently, several studies have reported that coercivity can be improved at high temperatures without the use of heavy rare-earth elements via alloy improvement [6] and process development [7,8], based on various advanced microstructural analyses [9].

The fabrication of high-performance permanent magnets capable of withstanding high temperatures is key to developing next-generation motors. Reported Sm–Fe-based compounds [10,11,12,13,14] exhibit a high Curie temperature of over 450 °C, and their anisotropic magnetic field and saturation magnetization are higher or comparable to those of Nd_2_Fe_14_B [1]. Sm–Fe-based compounds with intrinsic properties superior to those of Nd_2_Fe_14_B can be classified into two types based on their crystal structures: TbCu_7_ and ThMn_12_.

Compounds featuring the ThMn_12_ structure, such as Sm(FeCo)_12_ [15] and (SmZr)(FeCo)_12_ [16], are among the most promising permanent magnet materials reported to date, and their saturation magnetization at 300 K exceeds 1.7 T. However, because compounds with a ThMn_12_ structure and an Fe-rich composition that contain more Fe than the stable phase Sm_2_Fe_17_ are extremely unstable, reports of their bulk synthesis are lacking. For the bulk production of magnets with a ThMn_12_ structure, Fe should be substituted with a nonmagnetic structure-stabilizing element such as Ti, V, or Mo. However, when Fe is substituted, saturation magnetization is significantly reduced [17,18,19]. Recently, various studies have experimentally [14,20,21,22] and computationally [23,24,25,26] explored structurally stabilizing elements and their compositions while maintaining an Fe-rich Sm–Fe based composition.

Another Fe-rich Sm–Fe–N with a metastable phase and TbCu_7_-type structure possesses some of the best intrinsic properties among currently available bulk magnet powders, something which has recently been confirmed by epitaxial thin film experiments [27]. However, owing to the presence of the metastable phase, production remains possible only through the melt-spinning [12,13,28,29] and hydrogenation–decomposition–desorption–recombination processes [30,31,32,33]. Because only polycrystalline materials without a crystal orientation can be obtained using these processes, the intrinsic characteristics of the compound cannot be effectively utilized. Recently, Okada et al. reported a single-crystal, nanosized powder with a TbCu_7_-type phase prepared via low-temperature reduction diffusion [34,35]. While previous studies effectively promoted the reduction reaction only above the melting point of the Ca serving as a reducing agent [36], the authors realized the reduction reaction at a temperature lower than the melting point of Ca using molten salt, enabling the preparation of a single-crystalline powder with a metastable phase.

In this study, we focused on an induction thermal plasma (ITP) process as a breakthrough process for obtaining anisotropic nanosized powders of TbCu_7_-type Sm–Fe–N compounds. The thermal plasma processes are physical bottom-up processes characterized by quenching rates higher than that of the liquid-quenching method [37,38,39,40,41]. Thermal plasma is produced by an electric discharge at approximately 100 kPa; therein, atoms, ions, and electrons collide frequently. As a result, thermal plasma has high enthalpy and high flow speed [42,43,44]. By virtue of these features, material vapors can be transported and converted into nanosized powders rapidly in the flow field [45,46,47,48,49,50]. Although jet-type and arc-type thermal plasmas inherently include contamination from the electrodes, ITP is clean of contamination because the plasma is generated and sustained by electromagnetic induction without electrodes. Furthermore, the ITP has a region with a larger volume of high temperature, approximately 10,000 K, which achieves rapid evaporation of a large amount of raw precursor materials [51,52] and consequent high yields of powders [53,54,55,56,57]. Studies have demonstrated that a TbCu_7_-type compound can be obtained using the ITP process from the Nd–Fe [58] systems. These results indicate a high possibility of obtaining a TbCu_7_-type compound from the Sm–Fe system. Moreover, when the ITP process is applied, a fine permanent magnet anisotropic powder with a size of approximately 100 nm can be synthesized, which cannot easily be obtained using conventional breakdown processes such as jet-milling. This suggests that an anisotropic magnet with a large coercive force can be obtained. Therefore, in this study, we prepared a nanosized TbCu_7_-type Sm–Fe–N powder using the ITP process and evaluated its phase and magnetic properties.

This study is structured as follows: Section 2 describes the methods used for the preparation and characterization of the developed materials, as well as the numerical calculation methods. Section 3 presents the results and discussion based on the experimental and numerical results. Finally, Section 4 summarizes the paper’s references.

## 2. Materials and Methods

### 2.1. Preparation of the Raw Powder for ITP Process

**Figure 1:**
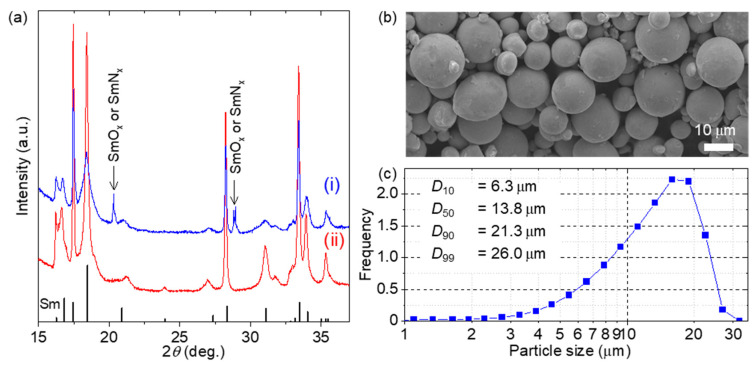
(**a**) XRD patterns of the Sm powder prepared by skull gas atomizing (i) with and (ii) without exposure to air. The energy of the incident X-ray is 14.00 keV (wavelength: 0.08857 nm). (**b**) SEM image and (**c**) particle size distribution of the skull gas-atomized Sm powder.

Because metallic Sm powder is not commercially available for use as a starting material, we prepared micron-sized Sm powder using a skull gas-atomizing device with a water-cooled copper crucible (CCGA-0.8, SINFONIA TECHNOLOGY Co., Ltd., Tokyo, Japan). To prevent the oxidation of the obtained Sm powder, it was handled without exposure to air. Figure 1a shows the X-ray diffraction (XRD) patterns of the Sm powders obtained (i) with and (ii) without exposure to air. No oxide peak was observed for the powder without exposure to air, whereas nitride and/or oxide peaks were detected for the powder exposed to air. Figure 1b shows the scanning electron microscopy (SEM) image of the Sm powder obtained by gas atomization. A highly spherical powder was obtained, which is a characteristic of the powder morphology obtained during gas atomization. In this experiment, nonexposed Sm powder was used as the starting material, and its particle size histogram is shown in Figure 1c. A mixture of Sm powder (*D*_90_ = 21.3 μm) and Fe powder (*D*_90_ = 6.7 μm, High Purity Chemical Laboratory, Saitama, Japan) with an atomic ratio of Sm:Fe = 1:x (x = 1.5 and 6.0) was used as the starting powder for the ITP process. Because the particle size of the Sm powder was relatively large for complete evaporation during the thermal plasma process, the starting ratio *x* was set larger than the target value of x = 9. The ITP process was performed at a process pressure of 100 kPa and an input power of 6 kW.

### 2.2. Characterization

The particle sizes of the coarse powders were measured using laser diffraction equipment (HELOS, Sympatec GmbH, Clausthal-Zellerfeld, Germany). The particle size of the ITP-processed nanopowder was estimated from the SEM image captured using JSM-7800S (JEOL Ltd., Tokyo, Japan) by fitting it to a log-normal distribution function. The nanopowder obtained using the ITP process was sealed in a quartz capillary with a diameter of 300 μm in a glovebox, and its crystal structure was evaluated by powder transmission XRD obtained at the BL5S2 beamline in Aichi Synchrotron Radiation Center (Aichi, Japan) at 14 keV without oxidizing the powder. In addition, a microstructural analysis was conducted using scanning transmission electron microscopy (STEM; JEOL Ltd., JEM-ARM200CF equipped with a CEOS ASCOR corrector) at an accelerating voltage of 200 kV. The powder was dispersed in an acetonitrile solution using a homogenizer and was then magnetically separated and oriented at 7 MA/m and 300 K in a resin. To obtain accurate information on its magnetic phase, magnetic separation was performed before magnetic field orientation to eliminate nonmagnetic compounds. The aligned sample was evaluated by a *θ*–2*θ* and a pole figure measurement at the Aichi BL8S1 beamline at 14.37 keV.

### 2.3. Numerical Calculation

To quantitatively evaluate the vapor-to-particle conversion and alloying processes, a numerical calculation was performed using the binary aerosol growth model with the nodal discretization technique [59]. The particles grew collectively, and their size and composition changed in a time-dependent manner. This process involves simultaneous homogeneous nucleation (metal vapor atoms form embryos of particles), heterogeneous condensation (metal vapor atoms meet and merge with embryos and particles), and interparticle coagulation (particles collide and unite) under the following assumptions:

• The particles are spherical.
• Bulk gas, metal vapors, and particles have the same temperature.
• Heat generation due to vapor condensation on particles is negligible.
• Metal vapor is considered an ideal gas owing to its high temperature.
• The melting point of particles depends on their size and composition [60,61] and is assumed to be uniform within the particle.
• The electric charge of particles is negligible under the present conditions.

It is noteworthy that charging effects can be considerable when many electrons exist in the region in which particles are generated and grow. For instance, electrons still remain even outside of plasma when the bulk gas and plasma contain species with low electron-recombination rates or when the spatial temperature gradient is very steep at the fringe of the plasma such as arc plasma [62]. The present process uses argon gas with a high electron-recombination rate and an ITP with relatively gentle temperature gradient, in which charging effects can be neglected.

**Figure 2:**
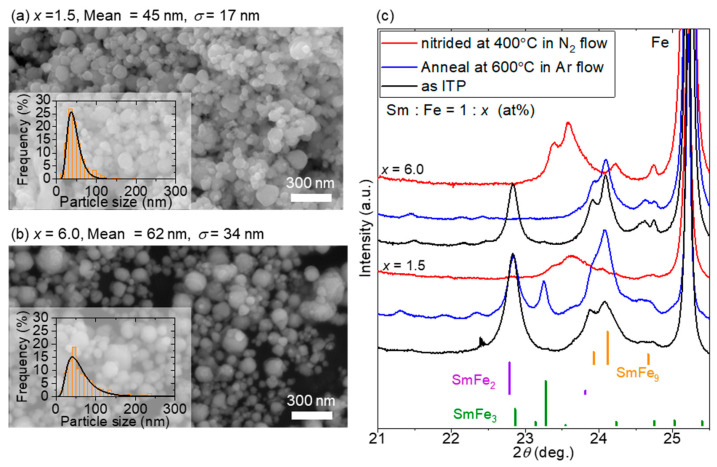
SEM images and histograms of the powder prepared via the ITP method at (**a**) *x* = 1.5 and (**b**) *x* = 6.0, respectively. The mean particle size and standard deviation *σ* are determined from the histograms by fitting them to the log-normal distribution function. (**c**) XRD patterns of the as-ITP-processed powder annealed at 600 °C in Ar flow and nitrided at 400 °C. The references of the peaks of TbCu_7_-type SmFe_9_, SmFe_2_ and SmFe_3_ are PDF#00-043-1311, PDF#00-025-1152 and PDF#00-050-1450, respectively. The energy of the incident X-ray is 14.00 keV (wavelength: 0.08857 nm).

The numerical calculation was performed under typical cooling conditions at the plasma tail, where the temperature monotonically decreased at a rate of 5.0 × 10^4^ K/s, which was determined to match the average particle size obtained from the experiment, as shown in Figure 2b. Under conditions identical to those in the experiment, the total feed rate of the precursory mixed powder with Sm:Fe = 1:9 (at %) was set to 0.25 g/min in the carrier Ar gas at 3.0 L/min. Detailed descriptions of the calculation conditions are provided in previous reports [58].

## 3. Results and Discussion

As estimated from the histograms obtained from the SEM images in Figure 2a,b, the ITP process produced nanopowders with mean particle sizes of 45 ± 17 and 62 ± 34 nm for *x* = 1.5 and 6.0, respectively. The mean particle size and standard deviation *σ* are determined from the histograms by fitting them to the log-normal distribution function. SmFe_2_ and Sm–Fe with TbCu_7_-type structures were observed in the samples with *x* = 1.5 and 6.0, respectively. After annealing at 600 °C, only Sm–Fe with the TbCu_7_-type structure was obtained for *x* = 6.0, while the *x* = 1.5 powder contained a sufficient amount of Sm to form Sm-rich compounds such as SmFe_2_ and SmFe_3_, as shown in Figure 2c. During the alloy formation in the ITP process, Sm condensation may be delayed compared with Fe condensation because of the significant difference in the vapor pressures and surface tensions of Sm and Fe (this formation process is discussed subsequently in the calculation). Therefore, atomic diffusion proceeded and resulted in the formation of alloy phases during heat treatment. This diffusion was also reported in the investigation of the obtained phases of the Sm–Fe system during low-temperature heat treatment [34].

When the lattice constant was estimated from the position of the XRD peak for the sample with *x* = 6.0 powder, *a* = 0.4935 nm and *c* = 0.4155 nm (*c*/*a* = 0.8419) were calculated for the sample before nitridation. Based on the lattice constant, the composition ratio (at %) of Sm and Fe in the TbCu_7_-type Sm–Fe compound prepared in this study is approximately 8.5 [12]. After nitriding by heat treatment at an elevated temperature of 400 °C in an N_2_ flow for 15 min, N was introduced into the Sm–Fe lattice with a TbCu_7_-type structure, as confirmed by lattice expansion. The introduction of N causes a lattice expansion of approximately 6.82% (*a* = 0.5057 nm, *c* = 0.4227 nm, *c*/*a* = 0.8359), indicating sufficient nitriding.

**Figure 3:**
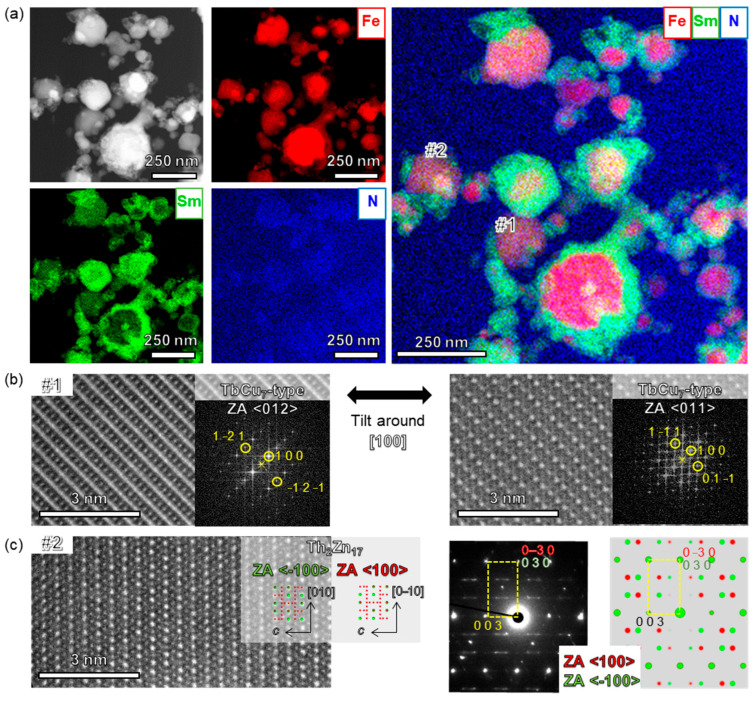
(**a**) Elemental maps for Sm, Fe, and N. (**b**,**c**) High-resolution STEM images of the #1 and #2 particles shown in (**a**), with FFT images and nanobeam diffraction only for (**c**).

Figure 3 shows the results of the microstructural analysis after nitriding with *x* = 6 Fe-rich compositions. The results of energy dispersive X-ray spectroscopy (EDX) show that Sm is highly concentrated on the particle surface and has a core–shell structure as shown in Figure 3a. In addition, sufficient N was present in the alloy, which was consistent with the XRD results. High-resolution STEM and fast Fourier transform (FFT) images for particle #1 are shown in Figure 3b. By tilting particle #1 around the [100] direction, two consecutive atomic images with zone axes of <012> and <011> were obtained, as shown in Figure 3b, indicating that the particle has a TbCu_7_-type crystal structure. Moreover, the particle is a single crystal. Thus, TbCu_7_-type single-crystal nanoparticles could be synthesized using the ITP process starting from the Sm–Fe and Nd–Fe systems [58]. However, particle #2 had a partially ordered Th_2_Zn_17_ structure, as shown in Figure 3c. Although this partially ordered Th_2_Zn_17_ structure is not as random as the TbCu_7_-type phase, two phases with a plane symmetry in the *y*–*z* plane of the Th_2_Zn_17_ phase were included. This crystal ordering fluctuation did not affect the magnetization because the *c*-axes of both TbCu_7_ and Th_2_Zn_17_ were the axes of easy magnetization. TbCu_7_ and Th_2_Zn_17_ cannot easily be distinguished from the microstructural observations of a single ZA. Therefore, as shown in Figure 3b, observation was performed from an appropriate ZA direction that can separate the disordered and ordered phases.

**Figure 4:**
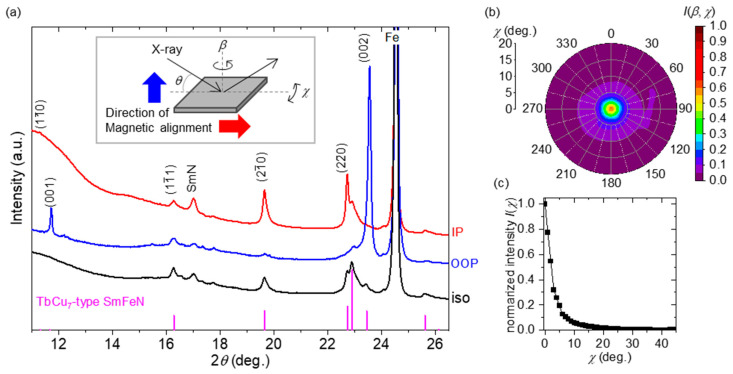
(**a**) XRD patterns of the nitrided sample of the Sm–Fe made with a ratio of Sm:Fe = 1:6, “iso” is a nonmagnetic-aligned sample provided for reference. The normal vector of the XRD measurement is parallel (OOP) or perpendicular (IP) to the direction of magnetic alignment. The reference of the peaks of TbCu_7_-type Sm_0.667_Fe_5.667_N_1.044_ is PDF#01-089-7982. The energy of the incident X-ray is 14.37 keV (wavelength: 0.0863 nm). The inlet figure shows the configuration of the angles of the *θ*/2*θ* and pole figure measurements. (**b**) Contour map of the count as a function of *β* and *χ*, and (**c**) normalized intensity profile that integrates *β* from 0 to 2π as a function of *χ*.

To prove that the TbCu_7_-type Sm–Fe–N nanoparticles were anisotropic, the powders were magnetically aligned at 9 T and 300 K in a resin, after which the aligned powers were examined using XRD at the aforementioned BL8S1 beamline at an X-ray energy of 14.37 keV (wavelength of 0.0863 nm). Figure 4a shows the *θ*–2*θ* patterns of the aligned powders, along with those of the nonaligned powders (“iso”) for reference. When the normal vector of the measurement surface is parallel to the direction of magnetic alignment, the dataset is denoted as “out of plane” (OOP), and other datasets in which the normal vector is perpendicular to the direction of magnetic alignment are denoted as “in plane” (IP). The intensity from the (0 0 *l*) plane was high in the OOP configuration, whereas the intensity from the (*h k* 0) plane was high in the IP configuration, indicating that the obtained nanoparticles were anisotropic and that the easy axis of the TbCu_7_-type Sm–Fe–N was the *c*-axis.

To quantify the degree of alignment of TbCu_7_-type Sm–Fe–N, pole figure measurements were conducted. The 2*θ* was fixed at a peak position of the (002) plane for the TbCu_7_-type structure, and the pole figure around the [001] direction was obtained by changing *b* and *c* at intervals of 3°, where *β* and *χ* are the angles, as shown in Figure 4a. The contour map of *I*(*β*, *χ*) is shown in Figure 4bc. High intensity was observed in the [001] direction (*β* = *χ* = 0). Figure 4c shows the intensity as a function of *χ* using Equation (1).

(1) $$I\left(\chi \right)=\int_{0}^{2\pi }I\left(\chi ,\beta \right)d\beta ,$$

Based on *I*(*χ*), the degree of alignment *P* was calculated using Equation (2).

(2) $$P\left[\%\right]=\left(2\times \frac{\int_{0}^{\pi /2}I\left(\chi \right)sin\chi cos\chi d\chi }{\int_{0}^{\pi /2}I\left(\chi \right)sin\chi d\chi }-1\right)\times 100,$$

where $\int_{0}^{\pi /2}I\left(\chi \right)sin\chi d\chi$ and $\int_{0}^{\pi /2}I\left(\chi \right)sin\chi cos\chi d\chi$ denote the total magnetic moment and the projection of the components of the total magnetic moment in the Z-direction along the external magnetic field, respectively [63]. Therefore, a *p* value of 91 ± 2% was estimated, indicating that the alloy nanoparticles with the TbCu_7_ structure had an extremely high orientation along the *c*-axis. To the best of our knowledge, a high degree of alignment for Sm–Fe–N with the TbCu_7_-type structure is experimentally demonstrated in this study for the first time.

**Figure 5:**
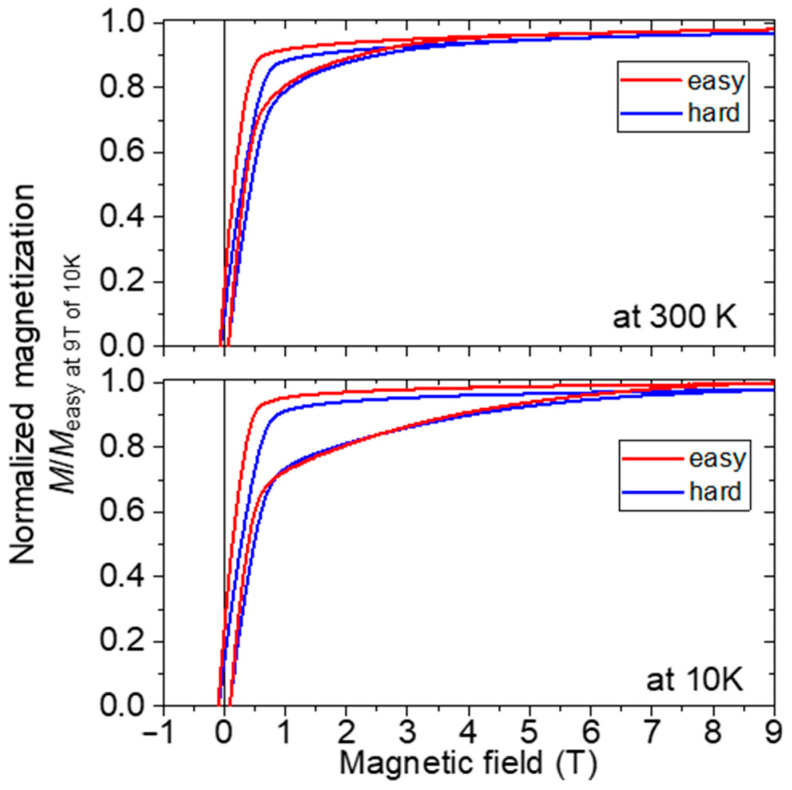
Hysteresis loops at 300 K (upper) and 10 K (lower) for the nitrided sample of Sm–Fe made with a ratio of Sm:Fe = 1:6. Configurations wherein the magnetic direction during the measurement is parallel and perpendicular to the magnetic alignment are denoted as “easy” and “hard,” respectively.

Figure 5 shows the measurement results at 300 and 10 K for the magnetic-field-oriented sample. The configurations in which the magnetic direction during the measurement is parallel and perpendicular to the magnetic alignment are described as “easy” and “hard”, respectively. The vertical axis was normalized by the magnetization obtained by the measurement in the easy axial direction at 9 T and 10 K. The difference between the hysteresis loops measured in the easy and hard directions can be observed at a low temperature of 10 K, where the anisotropic magnetic field increases. Therefore, the anisotropy field might be over 9 T at even 300 K. However, because this sample contained a considerable amount of Fe, the hysteresis curve differed from that of a compound with typical uniaxial anisotropy. Therefore, the magnetic anisotropy field cannot easily be determined accurately. Coercivity is extremely small, being 0.056 MA/m at room temperature and 0.08 MA/m at 10 K, while coercivity can increase when the amount of Fe existing in the core is reduced by controlling the cooling rate in the ITP process and/or post annealing. When the radius of the Fe core is small compared with the exchange coupling length of the Sm–Fe–N shell, a certain degree of coercivity can be maintained, but when the radius of the Fe core is long compared with the exchange coupling length of the Sm–Fe–N, the soft magnetic component of Fe has a large effect and the coercivity drops dramatically.

**Figure 6:**
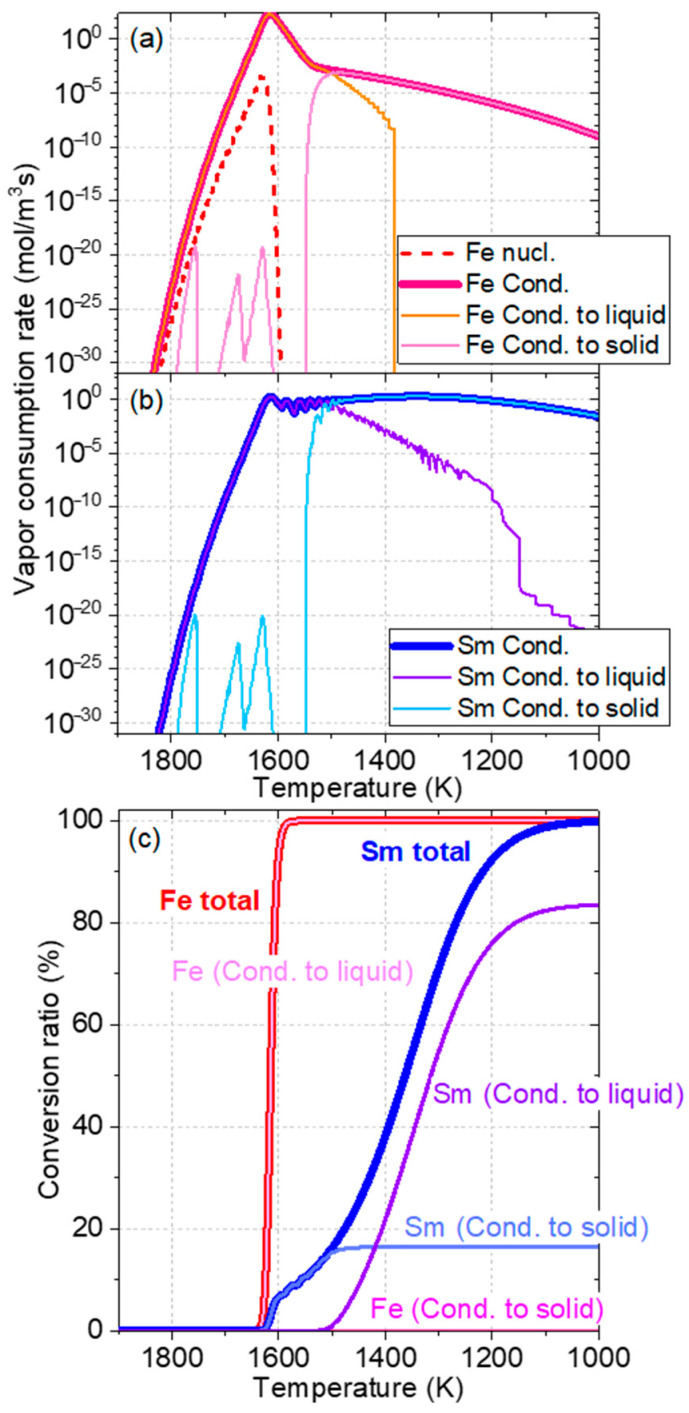
Vapor consumption rates of (**a**) Fe and (**b**) Sm. (**c**) Conversion ratios of Fe and Sm.

Figure 6 shows the results obtained from the numerical calculation of the vapor-to-particle conversion of the Sm–Fe particles. In Figure 6a,b, the condensation of both Fe and Sm starts simultaneously immediately after Fe nucleation occurs at approximately 1650 K. The maximum consumption rate of Fe is approximately 10^2^ times higher than that of Sm. This difference indicates that almost all of the Fe vapor is consumed at 1600 K, whereas only approximately 10% of the Sm vapor is consumed, as shown in Figure 6c. Thereafter, Sm vapor condensation continues up to 1100 K. From approximately 1500 K, the ratio of Sm vapor condensation to solid particles starts to increase, and, finally, approximately 80% of the Sm vapor condenses to solid particles. In this calculation model, Sm and Fe atoms mix uniformly in a particle, even if they condense into liquid and/or solid particles. The diffusion rates of Sm and Fe atoms in a particle are not considered. The particles obtained in the experiment were observed after a certain degree of alloying due to atomic diffusion. Notably, EDX images confirm the formation of Sm–Fe alloy particles with Sm-rich shells. Because Fe has similar intrinsic properties (vapor pressure and surface tension) to those of Co, the results obtained in this study show the same trends as those shown for the Sm–Co system [64]. Specifically, the nucleation temperature of Fe in the SmFe_7_ system is slightly lower than that of Co in the SmCo_5_ system, even with the same cooling rate of 5.0 × 10^4^ K/s, because of the Fe-rich composition.

**Figure 7:**
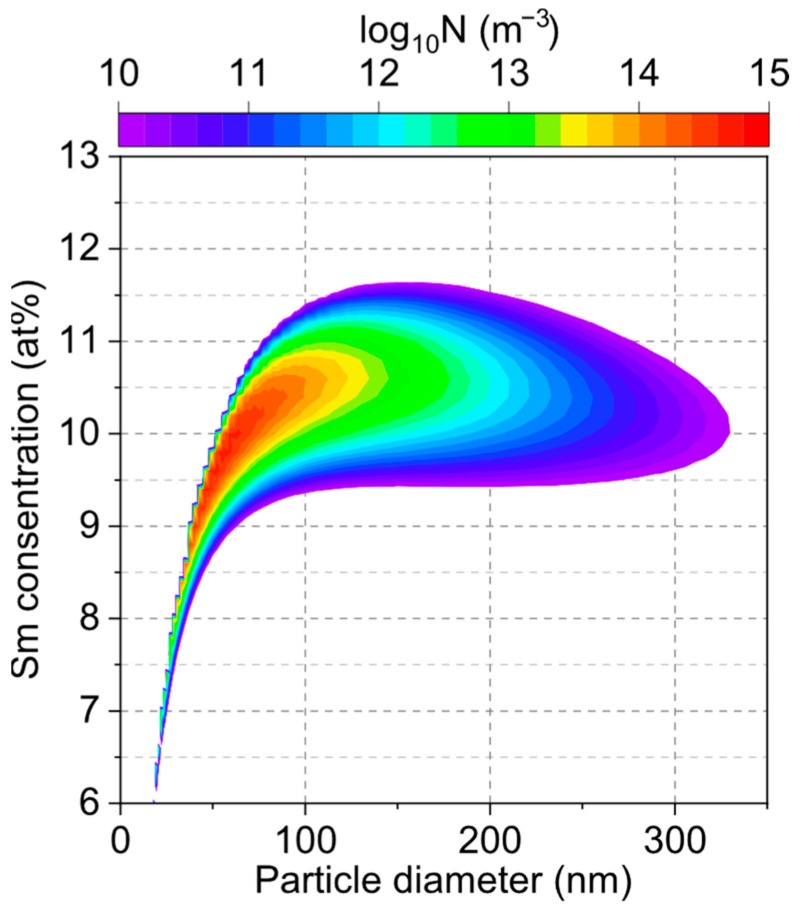
Particle number density map against the Sm concentration and particle diameter.

The present Sm–Fe system produces particles with a significant compositional distribution because the metal species pairs have significantly different saturation vapor pressures, as reported in the literature [65]. Figure 7 shows the final size and composition distributions of the particles at 300 K. This result indicates that the Sm concentration was within the range of 9–12 at%. Metastable TbCu_7_-type Sm–Fe can be synthesized within a range for Sm of up to 9.1(SmFe_10_)–12.5(SmFe_7_) at% [12,13]. Therefore, by sufficiently optimizing the process conditions, the ITP process can effectively obtain a single-phase TbCu_7_-type Sm–Fe alloy nanopowder. As a compound with high saturation magnetization has an Fe-rich concentration, a composition containing as much Fe as possible, such as an atomic ratio of Sm/Fe = 10, is necessary. An effective method for producing Fe-rich compounds is to substitute Zr or Y with Sm, which increases the c/a ratio and allows dumbbell Fe to be stably introduced into the lattice [13]. Therefore, In the future, we will attempt to enrich Fe by adding Zr and Y.

This study attempted to answer the question, “What type of particle formation process can be used to produce particles with a uniform composition?” This numerical calculation assumed a uniform composition inside each nanoparticle, which could differ from actual Sm–Fe nanoparticles. As aforementioned, in the case of Sm–Fe, approximately 10% of the particles pass through the liquid phase. Because the diffusion rate in the liquid phase is much higher than that in the solid phase, the advantage of passing through the liquid phase is significant for homogenizing the intraparticle composition. However, in Sm–Fe nanoparticles obtained by the solid-phase diffusion of Sm, if the diffusion length within a finite time is shorter than the particle size, core–shell particles with a Sm-rich phase are formed as the shell is formed. Therefore, to obtain a single-phase powder when the condensation temperatures are different, such as for Sm–Fe, the following conditions are necessary: (1) The time (temperature range) at which the droplets remained was expanded. However, the grain growth becomes significant as the cooling rate decreases [66]. (2) The solid-state diffusion time was prolonged. In the present Sm–Fe system, the residence time of many particles should be increased in the temperature range of 1100–900 K. However, conditions with a low cooling rate should be set during the ITP process. The realization of these conditions requires the control of heat flow at appropriate temperatures, and the results of numerical calculations significantly contribute to this realization.

## 4. Conclusions

In this study, a novel anisotropic Sm–Fe–N nanopowder with a TbCu_7_-type structure was prepared using the ITP process. The alloy phases of SmFe_2_ and TbCu_7_-type Sm–Fe compounds were obtained in the thermal plasma-processed state. In addition, Fe-core/Sm and/or Fe-core/SmFe-shell particles were observed using TEM. After nitriding, the TbCu_7_-type Sm–Fe–N and Fe phases were detected by XRD. Following magnetic alignment, the TbCu_7_-type Sm–Fe–N particles were well aligned along the *c*-axis, indicating that the TbCu_7_-type Sm–Fe–N compound exhibited uniaxial magnetic anisotropy. This anisotropy was confirmed by magnetic measurements. Although the magnetic properties of this material as a permanent magnet are still low because of the high Fe content, we synthesized a TbCu_7_-type Sm–Fe–N anisotropic powder with a particle size of less than 100 nm using a thermal plasma process. The atomic ratio of Fe/Sm was 8.5, indicating that an Fe-rich compound was not synthesized. In the future, we will attempt to enrich Fe by adding Zr and Y. The calculation results show that the vapor consumption of both Sm and Fe is a result of condensation, and that the condensation of Sm occurs at a significantly lower temperature than that of Fe. This is because of the difference in their saturation vapor pressures. Because the condensation of Sm occurs at low temperatures, approximately 90% of Sm merges with solid particles rather than with liquid droplets. This result is consistent with the TEM results, in which several particles with Sm-rich shells are observed. Therefore, to obtain Sm–Fe particles with a uniform composition within the particles, the temperature and time for diffusion should be determined, which is a guideline for future process improvements.

## Data Availability

Relevant data in this paper are available upon reasonable request.
